# Pencil Beam Scanning (PBS) Intensity-Modulated Proton Therapy (IMPT) Chemoradiotherapy for Anal Canal Cancer—Single Institution Experience

**DOI:** 10.3390/cancers14010185

**Published:** 2021-12-31

**Authors:** Pavel Vítek, Jiří Kubeš, Vladimír Vondráček, Michal Andrlik, Matěj Navrátíl, Radek Zapletal, Alexandra Haas, Kateřina Dědečková, Barbora Ondrová, Alexander Grebenyuk, Jozef Rosina

**Affiliations:** 1 Proton Therapy Center Czech, 180 00 Prague, Czech Republic; pavel.vitek@ptc.cz (P.V.); jiri.kubes@ptc.cz (J.K.); vladimir.vondracek@ptc.cz (V.V.); matej.navratil@ptc.cz (M.N.); radek.zapletal@ptc.cz (R.Z.); alexandra.haas@ptc.cz (A.H.); katerina.dedeckova@ptc.cz (K.D.); barbora.ondrova@ptc.cz (B.O.); 2 Department of Oncology, 2nd Faculty of Medicine, Charles University Prague and Motol University Hospital, V Úvalu 84, 150 06 Prague, Czech Republic; 3 Department of Health Care Disciplines and Population Protection, Faculty of Biomedical Engineering, Czech Technical University Prague, Sítná Square 3105, 272 01 Kladno, Czech Republic; jozef.rosina@lf3.cuni.cz; 4 Department of Health Protection and Disaster Medicine, Pavlov First Saint Petersburg State Medical University, Lva Tolstogo 6-8, 197020 Saint Petersburg, Russia; grebenyuk_an@mail.ru; 5 Department of Medical Biophysics and Informatics, 3rd Faculty of Medicine, Charles University, Ruská 87, 116 36 Prague, Czech Republic

**Keywords:** anal canal cancer, intensity modulated proton therapy, anal canal proton therapy

## Abstract

**Simple Summary:**

Eligible patients received PBS IMPT at a single institution. Treatment was administered in two volumes: 1—tumour with margins plus involved lymph nodes; 2—regional lymph node groups: perirectal (mesorectal), obturatory, inguinal, internal, external, and common iliac. The total doses of 57.5 GyE and 45 GyE, respectively, were administered in volumes 1 and 2 in 25 fractions, 5 fractions per week, respectively (a simultaneous integrated boost). Concomitant chemotherapy cisplatinum (CDDP) plus 5-FU or CDDP plus capecitabine was administered as per protocol. This single-institution study showed the high efficacy of PBS IMPT, achieving a high rate of complete regression. The 2-year overall survival, relapse-free survival and colostomy-free survival were 94.2, 93.8 and 91.0%, respectively. The haematological acute toxicity of grade 3–4 remained low. The acute toxicity completely resolved in all patients and had no lethal outcomes.

**Abstract:**

***Background:*** A favourable dose distribution has been described for proton beam therapy (PBT) of anal cancer in dosimetric studies. The relationship between dosimetric parameters in bone marrow and haematologic toxicity, treatment interruptions, and treatment efficacy has also been documented. There are only few references on clinical results of PBT for anal cancer. The primary objective of the retrospective study was to assess the efficacy of pencil beam scanning intensity-modulated proton therapy (PBS IMPT) in the definitive chemoradiotherapy of anal cancer. Secondary objectives were established to identify the risks of acute chronic toxicity risks and to assess colostomy rates. ***Materials and methods:*** Patients were treated for biopsy-proven squamous cell cancer (SCC) of the anus at initial or advanced stages. Eligible patients received PBS IMPT at a single institution. Treatment was administered in two volumes: 1—tumour with margins plus involved lymph nodes; 2—regional lymph node groups: perirectal (mesorectal), obturatory, inguinal, internal, external, and common iliac. The total doses of 57.5 GyE and 45 GyE, respectively, were administered in volumes 1 and 2 in 25 fractions, 5 fractions per week, respectively (a simultaneous integrated boost). Concomitant chemotherapy cisplatinum (CDDP) plus 5-FU or CDDP plus capecitabine was administered as per protocol. The treatment effect was assessed using DRE (digital rectal examination) and MRI (magnetic resonance imaging) within the follow-up period. Toxicity was scaled using CTCAE version 4.0 criteria. Results: 39 of 41 patients treated during the period of February 2014–August 2021 were eligible for analysis. All patients completed treatment, 76.9% without interruption. The median treatment time was 35 days (32–35). The median follow-up period was 30 months, 34 patients are alive to-date, 5 patients died prior to the date of analysis, and 2 deaths were unrelated to the primary disease. The 2-year overall survival, relapse-free survival, and colostomy-free survival were 94.2%, 93.8%, and 91.0%, respectively. Complete regression was achieved in 36 patients (92.3%), partial regression was achieved in 2 (5.1%), and immediate progression at end of treatment occurred in 1 patient (2.6%). Salvage resection was indicated for two patients in partial regression and due to severe chronic dermatologic toxicity. The grade 3 and 4 haematological toxicity rates were 7.7% and 5.1%, respectively. The most frequent non-haematological acute toxicities of grade 3–4 observed were dermatitis (23.1%), diarrhoea (7.7%), and dehydration (7.7%). Chronic toxicity emerged predominantly as skin atrophy/ulceration grade 2 (26.5%) and grade 3–4 (5.8%), and radiation proctitis grade 2 (38.2%) and grade 3 (2.9%). ***Discussion, conclusions:*** This single-institution study showed the high efficacy of PBS IMPT, achieving a high rate of complete regression. The haematological acute toxicity of grade 3–4 remained low; however, the impact of altered chemotherapy (CDDP instead of mitomycin C) remains unclear. The incidence of other acute toxicities shares similarity with photon therapy investigated in large studies. The acute toxicity completely resolved in all patients, had no lethal outcomes, and never resulted in the necessity for colostomy. By contrast, it was chronic toxicity, skin ulceration, perirectal fistulation, and fibrosis that resulted in salvage surgery and/or the need for a colostomy. A challenging question remains: to what extent can PBT prevent chronic toxicity? Longer follow-up remains necessary.

## 1. Introduction

The pioneering work of Nigro et al. [1] in 1974 first indicated that definitive radiotherapy with concomitant mitomycin C or porfyromycin had the potential to achieve a durable complete regression of anal canal cancer and was an effective alternative to radical surgery. However, a substantial acute and chronic toxicity was later documented in fundamental trials. The risk of acute toxicity of grade 3–4 in more than 85% of cases, as well as the risk of late toxicity grade 3–4 in 20% of cases, had been referred [2,3,4]. A significant improvement in toxicity was achieved when a more accurate technique of photon radiotherapy—intensity-modulated radiotherapy (IMRT)—was employed [5]. Naturally, proton radiotherapy, providing well-defined dosimetric advantages, was expected to decrease the incidence of acute and chronic side effects. A significant dose reduction in organs at risk was documented in initial dosimetric studies [6,7]. In addition to expectable dose reduction to the bladder, small intestine, and genitalia, there was a significant reduction in pelvic bone marrow. Other comparative studies have proved a dose distribution benefit, including that observed in bone marrow, compared to volumetric arc therapy (VMAT), IMRT, and 3D conformal radiotherapy techniques [8,9,10]. The results are important, since haematological toxicity may frequently result in treatment breaks and reduced efficacy [3,11].

A recent feasibility study indicated the efficacy of PBS IMPT for anal cancer in its initial and advanced stages [12]. Only acute toxicity analysis was published. Indirectly compared to RTOG 98-11 and RTOG 05-29 studies, the haematological toxicity of PBS IMPT was lower (however not the dermatological and gastrointestinal toxicity). The next series of phase II studies are underway and are soon to be completed [13,14]. Anal cancer is part of the portfolio of diagnoses treated at our institution. Ancillary dose distribution studies had favourable results, including a dose reduction in bone marrow, and were published in the form of an abstract [15]. The primary objective of this retrospective study was to assess the efficacy of pencil beam scanning proton radio-chemotherapy in the definitive treatment of anal cancer. A secondary objective was to evaluate both acute and chronic toxicity rates.

## 2. Materials and Methods

Patients were treated for anal cancer within the protocol of proton radiotherapy with concomitant chemotherapy. The study was approved by the institutional ethics committee and was conducted according to local ethical standards. All patients received PBS IMPT at a single institution.

Eligible patients were clinically fit, their ECOG performance status was 0–1, and all had biopsy-proven squamous cell cancer (SCC). The obligatory pre-treatment examinations included a digital rectal examination, an MRI of the pelvis, and a colonoscopy. ^18^FDG PET/CT was optional. Patients with metallic implants in the pelvic region, e.g., hip joint prosthesis, were ineligible for proton therapy due to dose calculation uncertainty.

### 2.1. Radiotherapy

PBS IMPT was administered in 2 treatment volumes:

− CTV-1: Tumour with a margin plus involved lymph nodes, total dose: 57.5 GyE.
− CTV-2: Regional lymph node groups—perirectal (mesorectal), obturatory, inguinal, internal, external and common iliac—total dose: 45 GyE.
− PTVs were generated by an expansion of 5 mm.
− Both doses in 25 fractions, 5 fractions/week, simultaneous integrated boost.

The dosage and fractionation were equal for all patients and TNM stages.

Treatment was administered in a prone or supine position. Whole-body fixation with a BlueBag (Elekta, Stockholm, Sweden) vacuum mattress was used for immobilisation in the initial period. Changes to Pelvicast (Orfit Industries, Wijnegem, Belgium) occurred in 2014. Planning CT was performed with a 2.5 mm slice distance. Treatment plans were calculated with the XiO treatment planning system (Elekta, Sweden) or RayStation treatment planning system (RaySearch, Stockholm, Sweden). The usual approach to planning was using 2–4 fields (dependent on the treatment volume). The robustness of treatment plans was assessed for one particular case with a typical setup. Consequent plans were constructed in a similar way, which allowed for the robustness to be considered equal. To account for possible uncertainties, a PTV concept was employed for planning. Tolerance doses for organs at risk were taken from the QUANTEC study. The following OARs were delineated in all patients: urinary bladder, femoral heads, external genitalia (penile bulb in males), small intestine, and sigmoid colon [15]. Priority in optimisation was always given to fulfilling OAR constraints over PTV coverage. CTV coverage (D_99_ = prescribed dose) was achieved in 95% patients. The lowest acceptable goal was to cover 98% CTV with at least 98% prescribed dose.

All patients underwent X-ray imaging in two orthogonal planes before each fraction. The position was corrected according to bone structure projection.

Regular CT simulation was performed to preclude gas filling of the sigmoideum and rectum, and to reduce dose uncertainties in intervals of 7 day or less, if necessary. QA plans were regularly calculated, and the replanning procedure followed in the case of substantial dose distribution disturbances.

### 2.2. Chemotherapy

All patients received concomitant chemotherapy—cisplatinum (CDDP) + 5-FU or CDDP + capecitabine at standard doses (CDDP 80 mg/m^2^ in the first and last week of radiotherapy, followed by 4 days of continuous infusion of 5-FU 1000 mg/m^2^/day or capecitabine 825 mg/m^2^ bid, for each day during the radiotherapy period, incl. weekends). Cisplatinum was considered an acceptable alternative to mitomycin C. There was no induction chemotherapy preceding radiotherapy.

### 2.3. Follow-Up

Throughout the radiotherapy course, patients were assessed weekly, and in the case of grade 3–4 side effects, more frequently. Essential biochemistry and blood examinations were performed weekly.

The treatment effect was assessed upon both DRE and MRI. Each complete or partial response had to be documented by both examinations. If tumour residue was apparent on DRE 8 weeks after treatment, the first post-treatment MRI imaging was delayed 3 months later.

In the case of rectal bleeding, a rectoscopy was performed to assess for signs of radiation proctitis.

Post-treatment check-up visits were scheduled for 8 weeks after therapy, then every 3 months subsequently. Each post-treatment visit included a digital rectal examination. MRI was planned annually for all patients, and in females, a gynaecological check-up was performed annually.

### 2.4. Statistics

Kaplan–Meier survival curves were calculated in order to determine survival outcomes (data analysis—MedCalc Software Ltd. 2021. Available online: https://www.medcalcsoftware.com/ (accessed on 15 October 2021).

Toxicity was scaled using CTCAE criteria version 4.0 for acute and late side effects.

The primary objectives included survival, relapse-free survival, and colostomy-free survival. The secondary objective of this analysis was to establish the limiting toxicity of PBS IMPT treatment, defined as grade 3–4 toxicity haematological toxicity below 20%, and other acute toxicity (GI, dermatological) below 25%. Chronic toxicity data were summarised but were not anticipated as conclusive.

## 3. Results

A total of 41 patients diagnosed with primary anal SCC were treated in the period of February 2014–August 2021. None of the patients were HIV positive. Chemoradiotherapy was administered as a frontline treatment in 39 of these cases and in another 2 as a treatment for localised relapse—therefore, they were excluded from any form of analysis. Patient characteristics are summarised in Table 1.

Radiotherapy was accomplished according to a plan for all patients. The median treatment time was 35 days (32–35). A total of 30 patients (76.9%) completed the radiotherapy course without interruption. There were interruptions demanded by toxicity in three patients (leukopenia and neutropenia gr. 4, hypokalaemia gr. 3) for a period of 3, 4, and 12 days, respectively. In the other six patients, treatment was interrupted for intercurrent complications (e.g., injury unrelated to the procedure), or for technical reasons. The median time of interruption was 7 days (3–20).

Concomitant chemotherapy (CDDP + 5-FU/CDDP + capecitabine) was administered in 33 patients (84.6%). Chemotherapy was omitted for various reasons such as age, comorbidity, stage T1N0M0, and refusal. Doses were reduced or the period of chemotherapy was shortened due to toxicity (haematological-only) in six patients (18%).

### 3.1. Treatment Efficacy

The median follow-up period was 30 months. A total of 34 patients remained alive at time of analysis, and 5 died prior to the date of analysis. Two deaths occurred and were unrelated to primary disease (heart failure, metachronous second primary—corpus uteri cancer 5 years post-treatment).

The 2-year overall survival, relapse-free survival, and colostomy-free survival rates were 94.2%, 93.8%, and 91.0%, respectively; see Table 2. Median values were not achieved.

**Figure 1:**
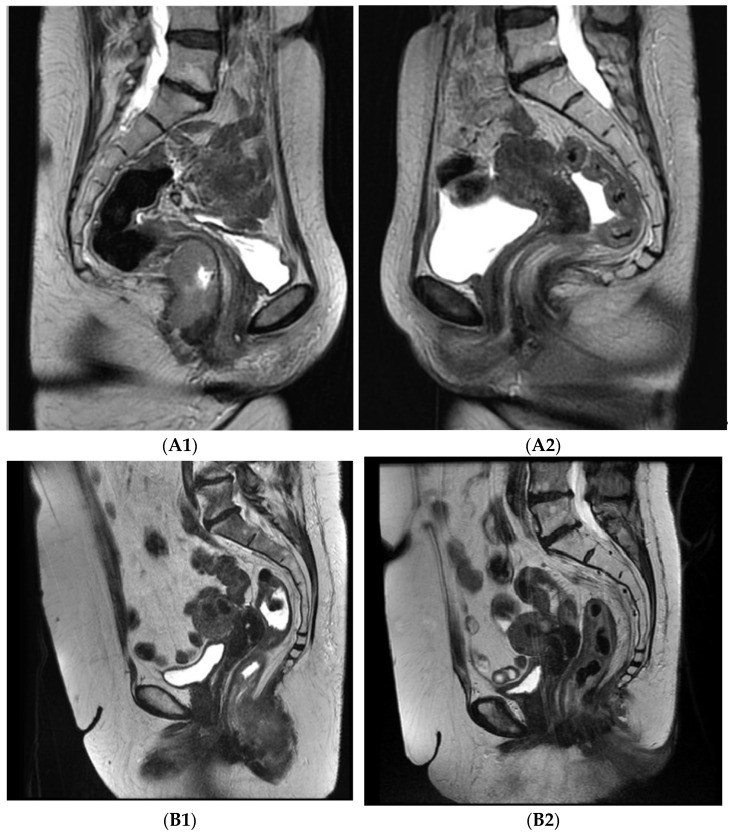
Axial T2 MRI images pre- and post-treatment: **A**—46 years old female, stage T3N0M0, (**A1**) pre-treatment, (**A2**) 8 weeks post-treatment, complete regression; **B**—62 years old female, stage T3N0M0, (**B1**) pre-treatment, (**B2**) 8 weeks post-treatment, partial regression.

Complete regression was achieved in 36 patients (92.3%), partial regression was achieved in 2 (5.1%), and immediate progression at end of treatment occurred in 1 patient (2.6%). Both patients with partial regression apparent on radiology imaging 5 months after treatment were referred to surgery, but one patient refused radical resection and preferred follow-up (Figure 1).

Metachronous relapse after complete regression was diagnosed in three patients—in two locoregional metastases and in one distant metastasis—9, 16, and 28 months after the commencement of treatment.

**Figure 2:**
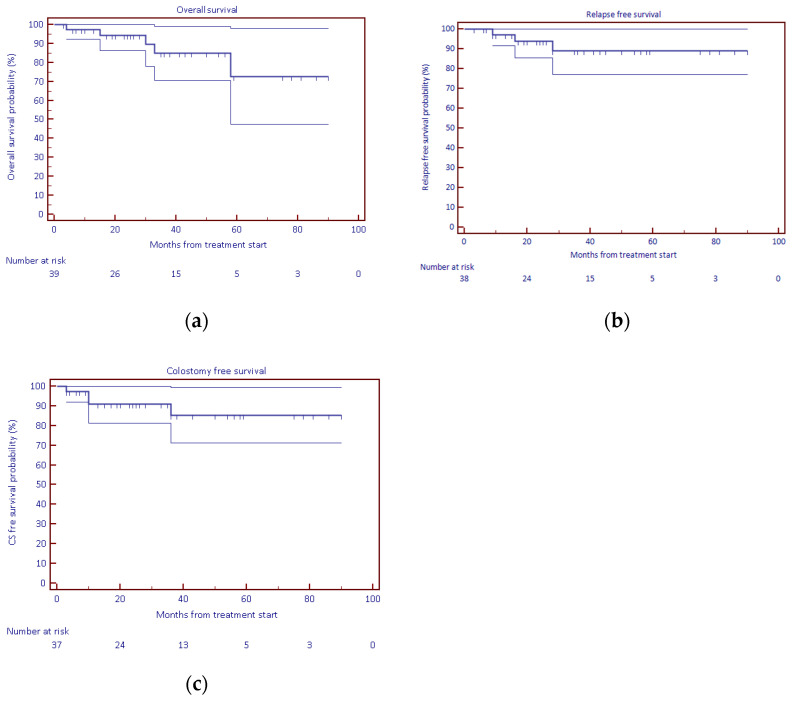
KM survival curves: (**a**) overall survival, (**b**) relapse-free survival, (**c**) colostomy-free survival.

Salvage surgery (abdominoperineal resection of the anus and rectum) was indicated in two patients—in one after partial regression of the initially voluminous pelvic mass, and in the other due to grade 3 dermatological late effects in the perianal region. Survival curves are presented in Figure 2.

### 3.2. Colostomy

Three patients underwent derivative colostomy prior to the commencement of treatment, and one was able to undergo colostomy reversal immediately following the termination of treatment. In the other three patients, colostomies were performed due to chronic toxicity complications: in two patients, derivative colostomies were performed, and in one patient, a terminal colostomy resulting from salvage surgery was performed (due to grade 3 skin late effects in the perianal region—see above).

### 3.3. Toxicity

The incidence of acute toxicity within the treatment period and 60 days following treatment is summarised in Table 3 and Table 4. Haematological toxicity resulted in treatment interruption in two patients for periods of 3 and 4 days.

The most frequent non-haematological cause of toxicity was dermal or mucosal in the perianal and anal (sphincter) regions. Locally managed with hygiene, washes, protection, and antiseptic measures, this did not result in interruptions to treatment.

No deaths occurred within the treatment period, or 60 days following treatment. Two substantial events occurred 1 week after the termination of radiotherapy—enterocolitis with the development of septic–toxic shock and paralytic bowel obstruction. Both complications resolved after conservative treatment. In general, all acute toxic complications resolved, and no long-term persisting consequences were observed.

Chronic toxicity (late effects) was assessed only in patients with a follow-up period longer than 6 months (34 patients). A summary of this is presented in Table 5. The most frequent chronic toxicity observed was dermatological—skin atrophy and telangiectasia. With the exception of one patient (originally with massive inguinal node involvement), only the perianal region was involved, with inguinal regions remaining unaffected. There were no functional consequences of skin toxicity, except for one case of grade 4 (ulceration combined with severe fibrosis) ultimately undergoing salvage surgery 3 years following treatment. All cases of radiation proctitis resolved after conservative (antiphlogistic or corticosteroid) treatment. Perianal fistulation developed in five patients, emerging as a result of disintegration of a large locally advanced (T3, T4) tumour involving the anal wall and adjacent tissues in two of the five patients. Perianal fistulation resulted in derivation colostomy in three of the five patients. One patient was able to undergo colostomy reversal after the fistula had regressed.

Anal stenoses did not require either salvage surgery or derivation and were sufficiently treated in a series of dilatations. Other anal functional impairments—dysreflexia, fractionated stools, and slight incontinence—were low grade and did not require derivation.

## 4. Discussion

Our single institutional results show the high efficacy of PBS IMPT—with a complete regression rate of 92.3%, as well as a favourable survival rate and a favourable colostomy-free rate. These effects were maintained despite 23.9% patients having interrupted treatment not due predominantly to toxicity. The efficacy of PBS IMPT is comparable with that observed in fundamental trials (ACT II, RTOG 98-11, RTOG 05-29) [2,3,5]. Proton therapy is undoubtedly non-inferior to photon radiotherapy techniques. The dose utilising protons was slightly higher; however, it remains questionable if this small dose increase could substantially impact efficacy. There was no intention to show a dose escalation effect, since data supporting the potential benefit of dose escalation are not available. Contrastingly, there are insufficient theoretical and dosimetric data supporting less toxicity when utilising PBT [6,7,8,9,10]. A significant dose reduction was documented in the pelvic organs—bowel; bladder; and genitalia, including the penile bulb. Moreover, a significant dose reduction to the bone marrow predicts a lower risk of haematological toxicity. Several studies have shown a direct relationship between dose parameters in bone marrow and haematological findings in peripheral blood within the context of photon radiotherapy [16,17,18]. In the case of proton therapy, a slightly lower haematological toxicity compared with RTOG 05-29 and RTOG 98-11 was documented in a recent study [12]. Conversely, the dermatological, gastrointestinal, and genitourinary toxicities remained within the same range. Additionally, our single-institution experience shows less haematological toxicity, especially a low incidence of grade 3–4 toxicity. Naturally, there is no direct comparison with photon therapy, and this can hardly be expected. However, these favourable results may confirm an expected projection of dosimetric advantage to clinical settings. How far our results were influenced by an alternative form of chemotherapy (CDDP instead of mitomycin C) remains unclear. Furthermore, non-haematological toxicity remains comparable to that observed in fundamental studies [2,3,5] in addition to recent proton experience [12]. Naturally, the low numbers of patients and various extents of the disease must be considered, as the patient characteristics are rather similar in recently referred groups, including our own—with the predominant characteristic being the T2N0 stage.

Of note, acute side effects were only a minor reason for treatment interruptions; acute toxicity completely resolved in all cases without any consequences, had no lethal outcomes, and did not result in colostomy. It is then questionable what is the priority of acute toxicity and how it impacts on the results of treatment. Answers may be provided by ongoing studies [13,14] using more advanced technologies. Acute toxicity does influence the feasibility of radiotherapy. Our data indicate the good feasibility of PBS IMPT and are in agreement with previously published data.

The current indicators of treatment quality (and efficacy to some extent), such as colostomy-free survival and functional consequences requiring surgery, are related to chronic toxicity rather than acute toxicity. In our experience, chronic toxicity resulted in three cases of colostomy and one of two cases of salvage surgery. It is confined to the skin, subdermal tissue, and possibly the rectum—there is no apparent relationship with radiation dose in bone marrow and other organs where the main dosimetric advantages of PRT are already well documented. We did not observe a high incidence of chronic toxicity; moreover, the most frequent observation was dermatological grade 2 toxicity—skin atrophy with telangiectasia without any functional consequences, and a negligible impact upon quality of life. However, the follow-up period was not long enough to regard the presented incidence of chronic side effects as conclusive. The drawbacks of the retrospective nature of this study must be accepted, especially the impact of the physician’s subjectivity regarding toxicity data. Moreover, it is rather difficult to assess quality of life unless validated questionnaires are provided to patients. The presented data show that the feasibility of PBS IMPT was good, and acute toxicity could be both decreased and surmounted. It is then necessary to assess how PRT contributes to prevent chronic side effects. In general, this presents a challenging issue due to the slow dynamics of late effects and an adequately long follow-up period. References to chronic effects are minimal in number in large studies, presenting a risk of about 20%. A significant impact on quality of life is evident in various analyses [19,20,21,22]. Moreover, there remains a question of how far chronic toxicity can be compared to the results of studies conducted many years ago (UKCCCR, RTOG 98-11) with dose distributions and technology that are hardly comparable [3,23]. If the incidence rates of chronic toxicity are simply numerically compared to our data (presumably anything beyond this is not possible), then it appears similar.

## 5. Conclusions

In conclusion, this single-institution experience documents the feasibility of PBS IMPT treatment for anal cancer. It shows high efficacy with a favourably low colostomy rate. Acute toxicity is moderate and does not result in significant treatment interruptions. Acute toxicity rates may be lower than in the case of conventional photon radiotherapy. The question remains as to how far PBS IMPT may reduce chronic toxicity, which results in major complications, including colostomies, and affects overall quality of life. Longer follow-up and more treatment are required to assess this observed benefit.

## Figures and Tables

**Table 1:** Patient characteristics.

Parameter	Nr.		Rem.
N	39		
Male	8	20.5%	
Female	31	79.5%	
Race	All Caucasian		
ECOG status 0	35	89.7%	
ECOG status 1	4	10.3%	
Age at diagnosis	Median 58 (41–82)		
Staging (all M0)			
T1N0	3	7.7%	
T2N0	14	35.9%	
T3N0	7	17.9%	
T4N0	1	2.6%	
T2N1	4	10.3%	
T3N1	3	7.7%	
T2N2	1	2.6%	
T4N2	2	5.1%	
T1N3	1	2.6%	
T2N3	1	2.6%	
T3N3	1	2.6%	
T4N3	1	2.6%	
Histology			
Spinocellular carcinoma	39		
Grade 1	5	12.8%	
Grade 2	15	38.5%	
Grade 3	8	20.5%	
Unknown	11	28.2%	
p16 positive/negative/not assessed	9/5/25	23.1%/12.8%/64.1%	
Synchronous malignancy	1		Synchronous uterine cervical cancer
Colostomy before radiotherapy	2		Derivative colostomy in locally advanced disease

**Table 2:** Efficacy data.

	Survival Proportion	Standard Error Confidence Interval 95%	
2-year overall survival	94.2%	4.0	90.2–98.2%
2-year relapse free survival	93.8%	4.3	89.5–98.1%
2-year colostomy free survival	91.0%	5.0	96.0–96.0%

**Table 3:** Acute haematologic toxicity (CTCAE version 4.0).

	Grade 1	Grade 2	Grade 3	Grade 4
Leukopenia	9 (23.08%)	7 (17.9%)	3 (7.7%)	2 (5.1%)
Neutropenia	1 (2.56%)	1 (2.6%)	3 (7.7%)	2 (5.1%)
Thrombocytopenia	1 (2.56%)	2 (5.1%)	0	0
Anaemia	8 (20.5%)	1 (2.6%)	0	0
Worst overall	10 (25.6%)	8 (20.5%)	3 (7.7%)	2 (5.1%)

**Table 4:** Acute non-haematologic toxicity (CTCAE version 4.0).

	Grade 1	Grade 2	Grade 3	Grade 4
Dermatitis	4 (10.3%)	24 (61.5%)	9 (23.1%)	0
Gastrointestinal				
Diarrhoea	1 (2.6%)	13 (38.2%)	2 (5.1%)	1 (2.6%)
Anal pain	1 (2.6%)	2 (5.1%)	2 (5.1%)	0
Proctitis	0	4 (10.3%)	0	0
Enterocolitis	0	0	1 (2.6%)	1 (2.6%)
Ileal obstruction	0	0	0	1 (2.6%)
Nausea	0	3 (7.7%)	0	0
Genitourinary				
Urinary tract pain	3 (7.7%)	4 (10.3%)	0	0
Urinary urgency	1 (2.6%)	2 (5.1%)	0	0
Other (general)				
Dehydration	0	2 (5.1%)	3 (7.7%)	0
Sepsis	0	0	0	1 (2.6%)
Other (laboratory)				
Transaminase (AST, ALT) increased	4 (10.3%)			
GGT, ALP increased	3 (7.7%)			
Creatinine increased	3 (7.7%)	3 (7.7%)	0	
Hypomagnesemia	3 (7.7%)			
Hypoalbuminemia	1 (2.6%)			
Hypokalaemia	0	2 (5.1%)	2 (5.1%)	1 (2.6%)

**Table 5:** Chronic toxicity (CTCAE version 4.0).

	Grade 1	Grade 2	Grade 3	Grade 4
Skin (perianal region)				
Skin atrophy	12 (35.3%)	9 (26.5%)	0	0
Telangiectasia	14 (41.2%)	0	0	0
Skin ulceration	0	0	1 (2.9%)	1 (2.9%)
Subcutaneous tissue fibrosis (perianal region)	4 (11.8%)	0	1 (2.9%)	0
Proctitis (post-radiation)	5 (14.7%)	13 (38.2%)	1 (2.9%)	0
Vaginal stricture (synechia)	0	3 (11.5%)	2 (7.7%)	0
Anal stenosis	4 (11.8%)	8 (23.5%)	1 (2.9%)	0

## Data Availability

The data presented in this study are available on request from the corresponding author.
